# Clinical Validation of the 2005 ISUP Gleason Grading System in a Cohort of Intermediate and High Risk Men Undergoing Radical Prostatectomy

**DOI:** 10.1371/journal.pone.0146189

**Published:** 2016-01-05

**Authors:** Sheila F. Faraj, Stephania M. Bezerra, Kasra Yousefi, Helen Fedor, Stephanie Glavaris, Misop Han, Alan W. Partin, Elizabeth Humphreys, Jeffrey Tosoian, Michael H. Johnson, Elai Davicioni, Bruce J. Trock, Edward M. Schaeffer, Ashley E. Ross, George J. Netto

**Affiliations:** 1 Department of Pathology, Johns Hopkins Medical Institutions, Baltimore, Maryland, United States of America; 2 Department of Urology, Johns Hopkins Medical Institutions, Baltimore, Maryland, United States of America; 3 Department of Oncology, Johns Hopkins Medical Institutions, Baltimore, Maryland, United States of America; 4 GenomeDx Biosciences, Vancouver, British Columbia, Canada; Sun Yat-sen University, CHINA

## Abstract

In 2005, the International Society of Urological Pathology (ISUP) introduced several modifications to the original Gleason system that were intended to enhance the prognostic power of Gleason score (GS). The objective of this study was to clinically validate the 2005 ISUP Gleason grading system for its ability to detect metastasis. We queried our institutional RP database for men with NCCN clinically localized intermediate to high-risk disease undergoing radical prostatectomy (RP) between 1992 and 2010 with no additional treatment until the time of metastatic progression. A case-cohort design was utilized. A total of 333 available RP samples were re-reviewed and GS was reassigned per the 2005 ISUP Gleason system. Cumulative incidence of metastasis was 0%, 8.4%, 24.5% and 44.4% among specimens that were downgraded, unchanged, had one point GS increase and two point GS increase, respectively. The hazard ratio for metastasis raised in GS 8 and 9 compared to GS 7 from 2.77 and 5.91 to 3.49 and 9.31, respectively. The survival c-index of GS increased from 0.70 to 0.80 when samples were re-graded at 5 years post RP. The c-index of the reassigned GS was higher than the original GS (0.77 vs 0.64) for predicting PCSM at 10 years post RP. The regraded GS improved the prediction of metastasis and PCSM. This validates the updated Gleason grading system using an unambiguous clinical endpoint and highlights the need for reassignment of Gleason grading according to 2005 ISUP system when considering comparisons of novel biomarkers to clinicopathological variables in archival cohorts.

## Introduction

Recent advances in our understanding of the molecular pathogenesis of prostate cancer have yielded promising prognostic molecular biomarkers and genomic assays [1–3]. Gleason score (GS) however remains one of the most powerful predictors of outcome in localized prostate cancer [4–8] and an essential element of risk stratification schemes [9].

The Gleason grading system was developed by Donald Gleason in 1966 [10] and subsequently slightly modified in the 1970s [11]. In 2005, the International Society of Urological Pathology (ISUP) introduced the first major modifications to the original Gleason system which was intended to further enhance the prognostic power of GS [5]. Several studies have established the validity of the 2005 ISUP modified Gleason systems in reaching a superior correlation between biopsy and radical prostatectomy (RP) as well as predicting biochemical recurrence (BCR) and outcome [12–14]. The 2005 ISUP modified GS is the basis for a proposed “Prognostic Grouping Scheme” soon to be adopted by the ISUP [8].

The objective of the current study is to clinically validate the ISUP 2005 Gleason grading system for its ability to predict the occurrence of BCR, metastasis, and prostate cancer specific mortality (PCSM) in a natural history cohort of intermediate and high risk men undergoing RP where all GS and pathologic features were reassigned by a central reviewer.

## Materials and Methods

This study was approved by the Institutional Review Board of Johns Hopkins University.

### Study Design

**Fig 1:**
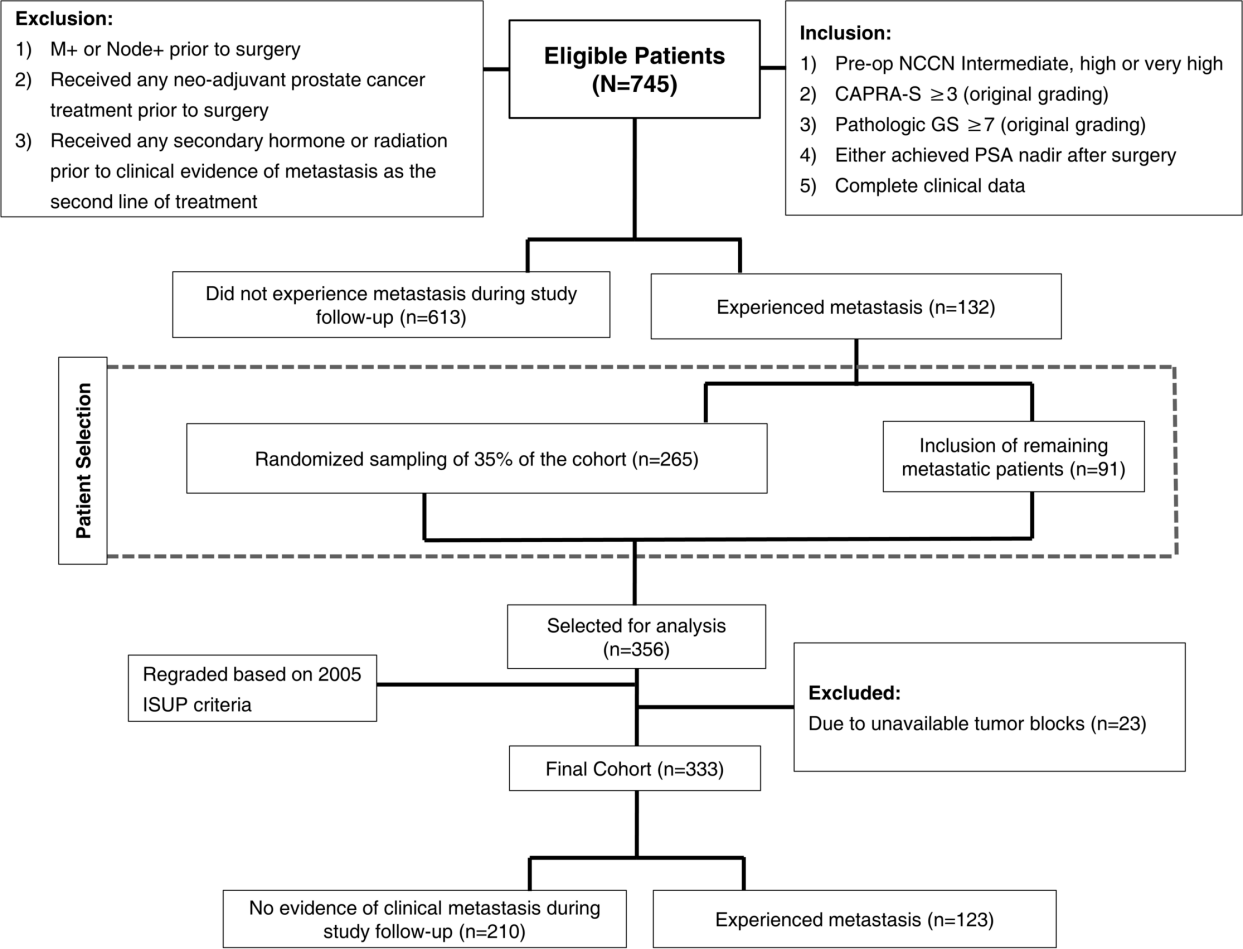
Study diagram and patient selection criteria.

Prostate cancer patients treated with RP between 1992 and 2010 were queried from our institutional database. This involved identification of all patients who met the following criteria: pre-operative NCCN intermediate (cT2b-T2c or GS 7 or PSA 10–20 ng/ml), high (cT3a or GS 8–10 or PSA > 20ng/ml) and very high (multiple high risk features, primary pattern 5 in any core, more than 4 cores with GS 8–10, T3b-4) risk disease [15] and had CAPRA-S scores of ≥3. In order to examine the effect of utilizing the updated ISUP on clinical progression, we only selected patients who had achieved PSA nadir after surgery defined as PSA < 0.2 ng/ml or had pre-operative imaging confirming local disease only (patients with metastatic disease and positive lymph nodes prior to surgery as well as the ones who received any neoadjuvant treatment prior to surgery were excluded). In addition, to examine the utility of the current Gleason grading system we limited our study to a natural history cohort and thus excluded men who received hormone, chemotherapy, or radiation therapy prior to clinical evidence of metastasis. Clinical metastasis was defined as a positive imaging study (i.e. by bone scan, CT scan or MRI). A total of 745 patients met the inclusion/exclusion criteria. A case-cohort design was used to randomly select 35% (n = 265) of the patients from the entire cohort. Following the case-cohort design, 91 non-selected metastatic patients were added to the randomly selected sub-cohort [16]. Due to lack of available sections and paraffin blocks, 23 patients were excluded from the 356 patients that were initially selected for analysis. A total of 333 men formed the final cohort of the study and grouped as follow: i) men who developed metastasis following RP (n = 123) and ii) men who did not experience clinical evidence of recurrence during study follow-up (n = 210, Fig 1). All RP slides were reviewed by two expert urologic pathologists (GJN in consensus with SMB or SFF), blinded to patient outcome, for GS reassignment according to 2005 ISUP modified Gleason System [5] and assessment of extraprostatic extension, seminal vesicle (SV) invasion and surgical margin status. Reviewed 2005 ISUP modified GS was compared to the original GS.

### Statistical Analysis

Primary endpoint of the study was defined as metastases evidenced by axial imaging or nuclear medicine bone scan. Biochemical recurrence and PCSM were considered as secondary endpoints. Upgrading was defined as at least one point rise in GS while downgrading was defined as at least one point decrease in GS.

Cumulative incidence curves for original and re-graded GS were constructed to estimate the risk of metastasis. These curves were obtained after reweighting the controls by inverse of the sampling fraction as described by Barlow et al [16] to take case-cohort design of the study into account. Discrimination was measured by concordance index for survival data at 5 years post RP [17]. Net benefit was estimated using extension of decision curve analysis to survival data at 5 years post RP [18]. Univariable and multivariable Cox proportional-hazards regression was performed to evaluate the prognostic ability of original and re-graded GS using the “Lin-Ying” method for the case-cohort design [19]. The significance level was 0.05 for all statistical tests and analyses were performed in R v3.1 (R Foundation, Vienna, Austria).

## Results

**Fig 2:**
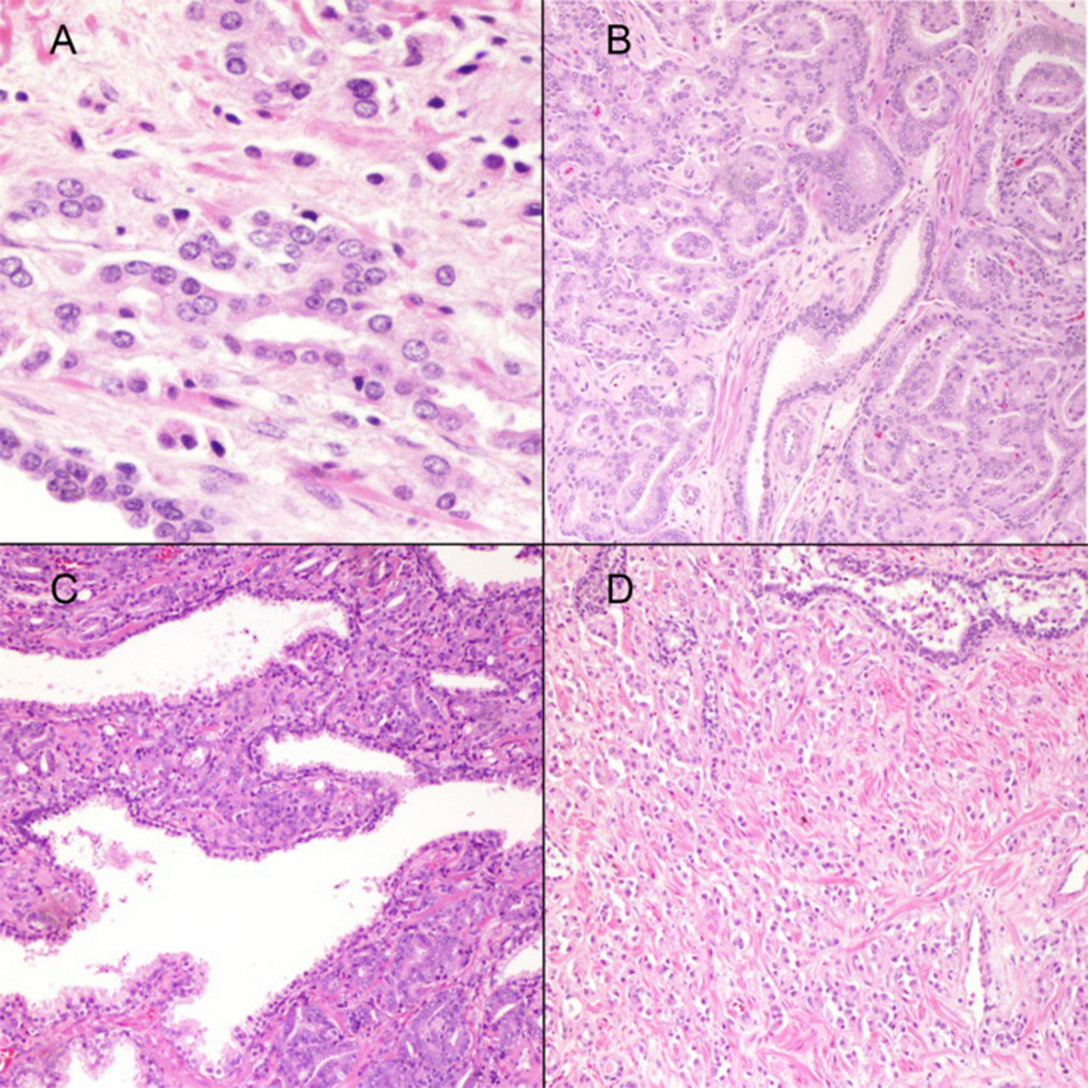
(A) Fused glands (400x). In the original Gleason grading this pattern was considered pattern 3. According to the 2005 ISUP Gleason system it would be graded as pattern 4; B) Adenocarcinoma with glomeruloid features currently assigned a Gleason pattern 4 (100x); C) Ill-defined glands (100x). This pattern would be graded as Gleason pattern 4 by the ISUP 2005 Gleason grading; and D) Individual cells (100x). This pattern was originally accepted under Gleason pattern 3 and would be assigned a Gleason pattern 5 according to the 2005 ISUP Gleason system.

Patient demographics and pathologic specimen characteristics are summarized in Table 1. Selected examples of reassigned GS are depicted in Fig 2.

**Table 1:** Cohort Demographic and Clinical Characteristics.

Variables	Validation Cohort (N = 333)
**Race, n (%)**	** **
Caucasian	299 (89.8%)
African-American	26 (7.8%)
Asian	0 (0%)
Other	5 (1.5%)
Unknown	3 (0.9%)
**Patient age, yr**	** **
Median (Range)	60 (38, 72)
IQR (Q1, Q3)	56–64
**Year of surgery**	** **
Median (Range)	1997 (1992, 2010)
IQR (Q1, Q3)	1994–2001
**Preoperative PSA (ng/ml)**	** **
Median (Range)	10.2 (1.79, 79.09)
IQR (Q1, Q3)	6.6–15.7
**Biopsy Gleason Score, n (%)**	** **
≤6	102 (30.6%)
7	172 (51.7%)
8	41 (12.3%)
≥9	18 (5.4%)
**Original Gleason Score, n (%)**	** **
3+4	129 (38.7%)
4+3	63 (18.9%)
8	39 (11.7%)
≥9	102 (30.6%)
**ISUP Regraded Gleason Score, n (%)**	** **
3+4	146 (43.8%)
4+3	73 (21.9%)
8	58 (17.4%)
≥9	56 (16.8%)
**Pathology Weight (grams)**	** **
Median (Range)	53.8 (25, 150)
IQR (Q1, Q3)	45–63
**Extraprostatic Extension, n (%)**	** **
	240 (72.1%)
**Seminal Vesicle Invasion, n (%)**	** **
	88 (26.4%)
**Positive Surgical Margins, n (%)**	** **
	91 (27.3%)
**Lymph Node Invasion, n (%)**	** **
	64 (19.2%)
**Follow up for censored patients, yr**	
Median (Range)	10 (5–19)
IQR (Q1, Q3)	6–13

Abbreviations: IQR = interquartile range, mo = months, PSA = prostate specific antigen, RP = radical prostatectomy, yr = year

### Comparison of original and reviewed radical prostatectomy pathologic parameters Gleason score

**Fig 3:**
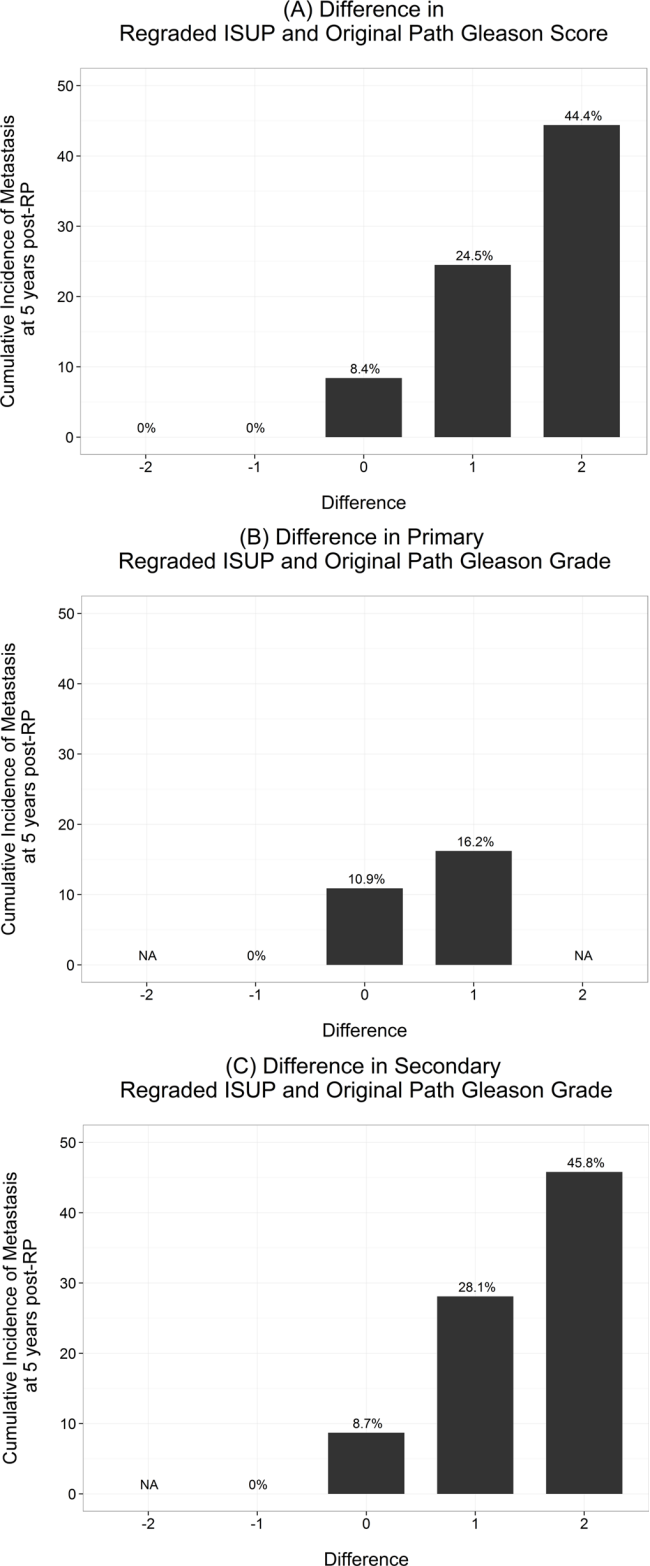
Cumulative incidence of metastasis at 5 years post radical prostatectomy. A) Difference in re-graded 2005 ISUP modified and original Gleason score; B) Difference in primary re-graded 2005 ISUP modified and original Gleason score; and C) Difference in secondary re-graded 2005 ISUP modified and original Gleason score.

Differences between reassigned ISUP 2005 GS and original GS are graphically illustrated in Fig 3. Cumulative incidence of metastasis was 0%, 8.4%, 24.5% and 44.4% among specimens that were downgraded, unchanged, had one point GS increase and two point GS increase, respectively. Table 2 shows that 57 of 333 patients had changes in GS; 37 of which were among the group who developed metastasis. Most of changes were due to changes in secondary Gleason pattern (Fig 3B and 3C).

**Table 2:** Changes in regraded variables compared to original variables by oncologic outcome.

Variables	Non-Metastatic*	Metastatic	All Patients
Change in Gleason Score, n	20	37	57
Change in Primary Gleason Grade, n	13	7	20
Change in Secondary Gleason Grade, n	24	36	60
Change in Surgical Margin Status, n	6	12	18
Change in Extraprostatic Extension, n	13	15	28
Change in Seminal Vesicle Invasion, n	7	3	10

*These patients did not metastasize during study follow-up.

### Extraprostatic extension, seminal vesicle invasion and margins status

Table 2 summarizes changes in reviewed compared to original variables by oncologic outcome. Surgical margins status was changed in 18 cases, 12 of which in metastatic patients. Extraprostatic extension changed in 15 metastatic and 13 non-metastatic patients. Changes in SV invasion occurred in 7 non-metastatic patients while 3 metastatic changed.

### Association of original Gleason Score and reassigned ISUP 2005 Gleason Score with Patient Outcome

**Fig 4:**
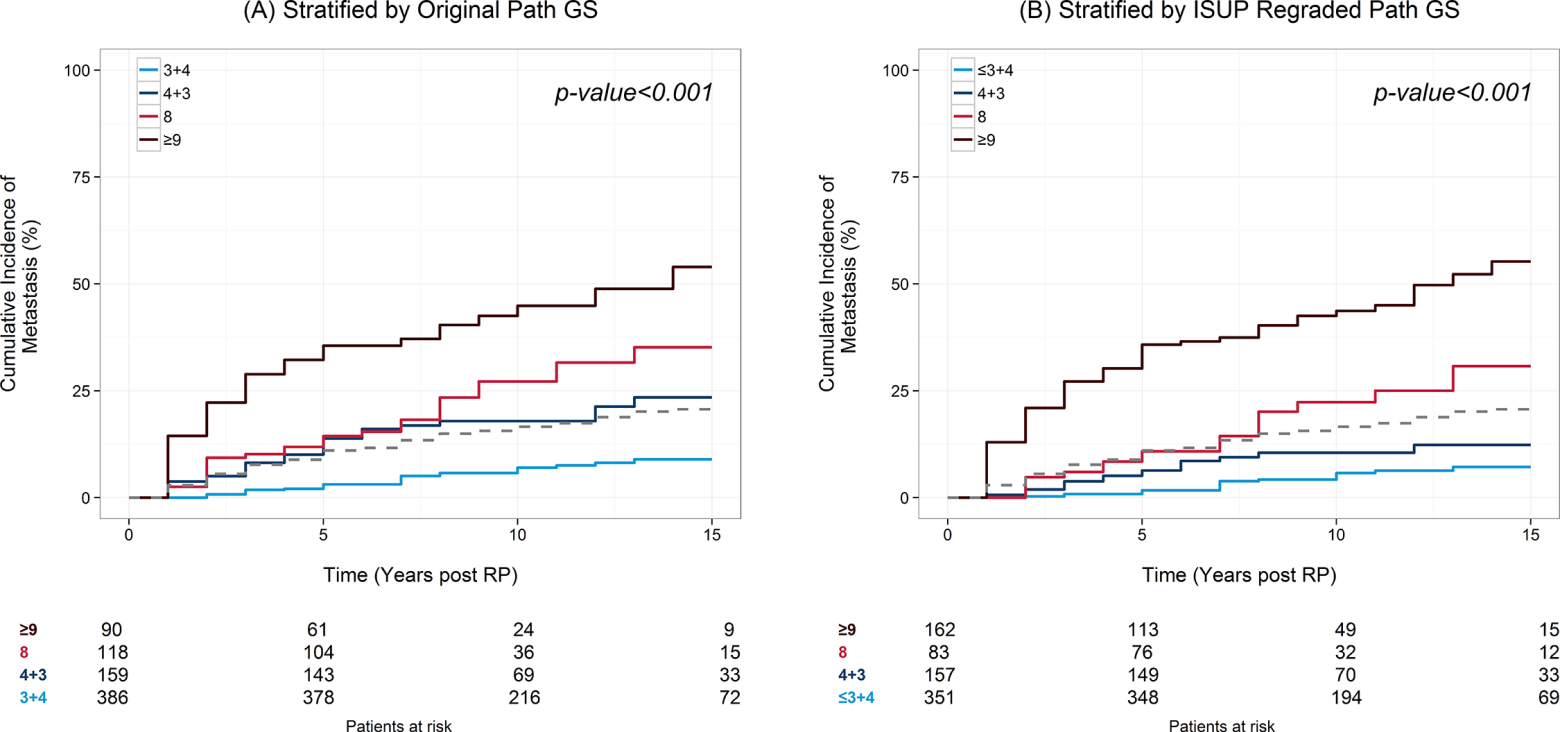
Cumulative incidence curves of A) metastasis by original Gleason score, B) metastasis by reviewed Gleason score. The number at risk in each group is shown in the footnote lines. Patients in the sub-cohort who do not experience the event are weighted by the inverse of the sampling fraction in following the case-cohort design.

Cumulative incidence curves for the development of BCR, metastasis, and PCSM were stratified by original GS as well as re-graded ISUP 2005 GS as shown in Fig 4 and S1 Fig. As expected, both original and re-graded ISUP Gleason scores were prognostic of prostate cancer outcomes. ISUP re-grading increased the number of cases classified as GS 9 prostate cancer. These reclassified cases had similar outcomes to GS 9 cancer that was not reclassified (data not shown) and were responsible for a better stratification of oncologic outcomes by Gleason grade.

Univariable and multivariable Cox regression analyses of two models based on original (model 1) and reviewed pathologic parameters (model 2) are summarized in Table 3. On univariable analysis, the reassigned ISUP 2005 GS better predicted the development of metastasis post RP. Compared to the reference GS of ≤7, the hazard ratio (HR) for association between GS 8 and development of metastases increased from 2.77 (95% confidence interval [CI]: 1.62–4.73) for the original GS to 3.49 (95% CI: 1.81–6.75) when the modified ISUP 2005 GS was used. The HR for association between GS 9 and metastasis increased from 5.91 (95% CI: 3.46–10.08) for the original GS to 9.31 (95% CI: 5.74–15.11) for the reassigned ISUP 2005 GS.

**Table 3:** Univariable and Multivariable Cox proportional hazards analysis of risk factors using original and ISUP regraded GS.

	Variables	UVA		MVA	
		HR (95% CI)	p-value	HR (95% CI)	p-value
**Model 1**	Patient age, yr	0.99 (0.96–1.02)	0,64	1.02 (0.98–1.06)	0,34
	Log2 Preoperative PSA (ng/ml)	1.20 (0.92–1.57)	0,17	1.12 (0.87–1.45)	0,39
	Original GS 7	ref	1	ref	1
	Original GS 8	2.77 (1.62–4.73)	<0.001	2.14 (1.06–4.32)	0,034
	Original GS 9	5.91 (3.46–10.08)	<0.001	5.2 (3.05–8.85)	<0.001
	Original Extraprostatic Extension	7.04 (3.17–15.60)	<0.001	5.71 (2.57–12.68)	<0.001
	Original Seminal Vesicle Invasion	7.60 (4.83–11.95)	<0.001	3.79 (2.29–6.26)	<0.001
	Original Positive Surgical Margins	2.09 (1.35–3.23)	<0.001	1.8 (1.14–2.84)	0,012
	Lymph Node Invasion	6.74 (4.21–10.81)	<0.001	2.7 (1.56–4.68)	<0.001
**Model 2**	Patient age, yr	0.99 (0.96–1.02)	0,64	1.01 (0.97–1.04)	0,76
	Log2 Preoperative PSA (ng/ml)	1.20 (0.92–1.57)	0,17	1.05 (0.82–1.34)	0,71
	ISUP GS 7	ref	1	ref	1
	ISUP GS 8	3.49 (1.81–6.75)	<0.001	2.07 (0.91–4.74)	0,085
	ISUP GS 9	9.31 (5.74–15.11)	<0.001	5.61 (3.34–9.43)	<0.001
	Extraprostatic Extension	4.03 (2.22–7.34)	<0.001	1.56 (0.78–3.14)	0,21
	Seminal Vesicle Invasion	8.08 (5.14–12.70)	<0.001	3.54 (2.06–6.08)	<0.001
	Positive Surgical Margins	2.10 (1.36–3.25)	<0.001	1.72 (1.05–2.82)	0,033
	Lymph Node Invasion	6.74 (4.21–10.81)	<0.001	2.97 (1.66–5.31)	<0.001

Abbreviations: CI = confidence intervals, HR = hazards ratio, GS = Gleason score, PSA = prostate specific antigen, yr = year

Presence of extrapostatic extension, SV invasion and positive surgical margins was also significantly associated with development of metastases using either original or reviewed parameters. On multivariable analysis, GS, presence of SV invasion, positive surgical margin and lymph node metastasis were significantly associated with metastasis in both models. On the other hand, presence of extraprostatic extension was only significant in model 1.

On additional univariable analysis, the HR for association between PCSM and original GS was 3.96 (95% CI: 1.47–10.66; p = 0.006) and 2.79 (95% CI: 0.75–10.36; p = 0.12) for GS 8 and GS ≥ 9 compared to GS 7 as reference, respectively. For association between PCSM and re-graded GS, the HR was 2.52 (95% CI: -.49–12.98; p = 0.27) and 9.51 (95% CI: 3.33–27.07; p<0.001) for GS 8 and GS ≥ 9, respectively.

**Fig 5:**
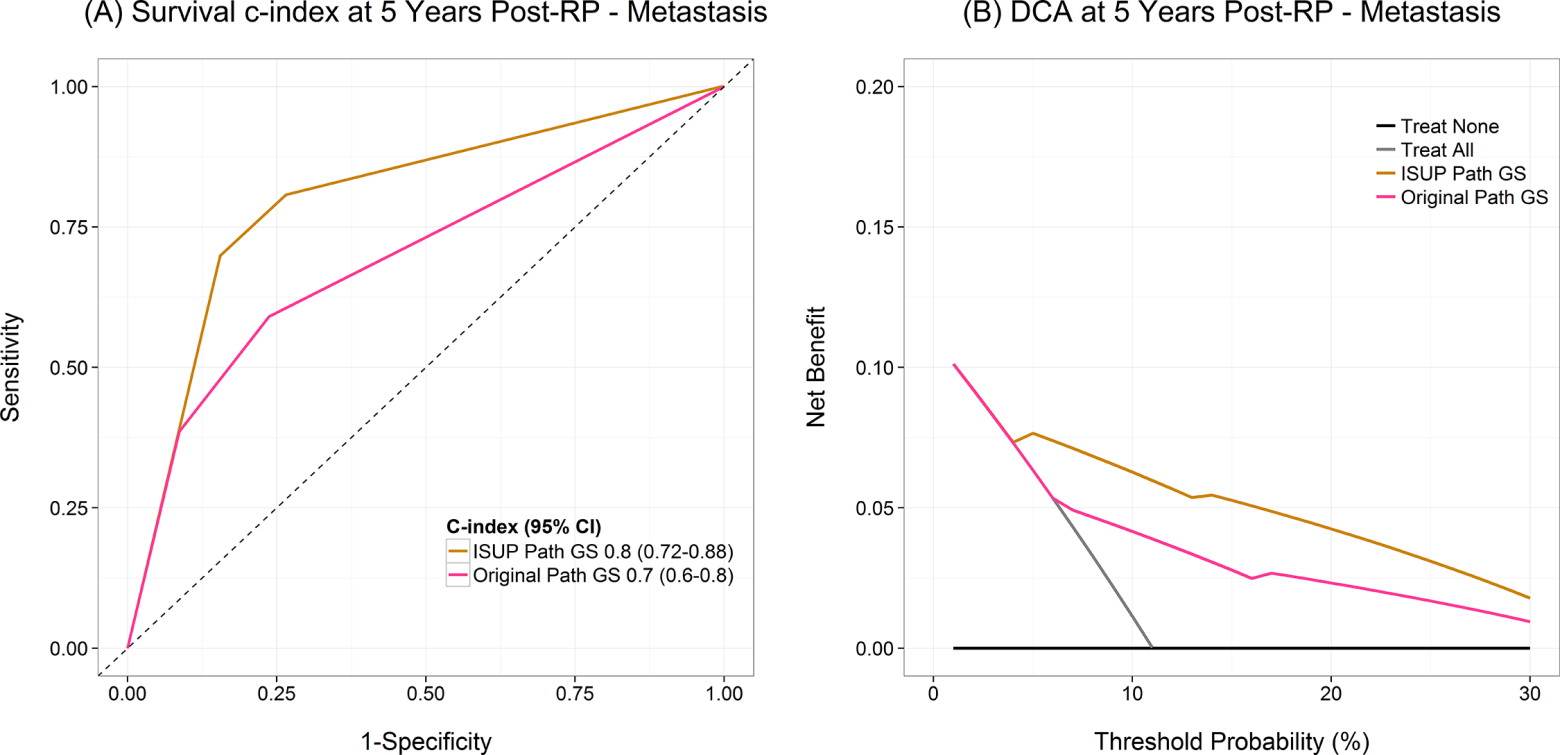
A) Survival concordance index. Reviewed Gleason score by the 2005 ISUP modified Gleason system has the highest c-index compared to original Gleason score. B) Decision curve analysis at 5 years post radical prostatectomy shows the net benefit of original and reviewed Gleason score across probability thresholds. The reviewed Gleason score shows the highest net benefit.

Survival concordance index and decision curve analysis findings are depicted in Fig 5. As shown in Fig 5A, the reassigned ISUP 2015 GS outperformed the original GS in survival concordance index at 5 years post RP (C-index: 0.80 [95% CI: 0.72–0.88] vs 0.70 [95% CI: 0.60–0.80]). Likewise, the reassigned ISUP 2005 GS showed a superior net benefit on decision curve analysis at 5 years post RP across a wide range of probability thresholds from ~ 5–30% (Fig 5B). The concordance index of the reassigned GS was higher than the original GS (C-index: 0.77 [95% CI: 0.69–0.92] vs 0.64 [95% CI: 0.56–0.84]) for predicting PCSM at 10 years post RP. On decision curve analysis on PCSM at 10 years post RP, reassigned ISUP 2005 showed higher net benefit compared to original GS (S2 Fig).

Finally we explored whether pathologic ISUP re-grading would modulate the utility of molecular tissue testing. 260 cases of the current cohort were previously analyzed by Decipher [2]. In a multivariable analysis, HRs for metastasis post-RP for Decipher were 1.42 (95% CI: 1.24–1.64; p<0.001) and 1.28 (95% CI: 1.08-1-53; p = 0.005) when adjusting for original and re-graded GS, respectively (Table 4).

**Table 4:** Multivariable Cox proportional hazards analysis of Decipher adjusting for original and ISUP regraded GS.

	Variables	MVA	
		HR (95% CI)	p-value
**Model 1**	Original GS 7	ref	1
	Original GS 8	1.32 (0.68–2.55)	0,41
	Original GS 9	4.12 (2.14–7.93)	<0.001
	Decipher*	1.42 (1.24–1.64)	<0.001
**Model 2**	ISUP GS 7	ref	1
	ISUP GS 8	1.74 (0.75–4.01)	0,19
	ISUP GS 9	6.67 (3.68–12.1)	<0.001
	Decipher*	1.28 (1.08–1.53)	0,005

Abbreviations: CI = confidence intervals, HR = hazards ratio, GS = Gleason score, PSA = prostate specific antigen, yr = year

*Decipher is reported per 0.1 unit increase

## Discussion

Since its original description, several aspects of the Gleason grading system have changed where morphologic patterns would be interpreted differently in contemporary pathology practice. The most salient modifications were introduced by the 2005 ISUP consensus conference which led to the adoption of the “2005 ISUP Modified Gleason Grading System” by the pathology community [5–8]. In addition to the virtual absence of Gleason patterns 1 and 2 in current practice, the most prominent changes include the expanded assignment of pattern 4 to almost all cribriform gland patterns as well as fused and poorly formed glands. Individual cells originally accepted under Gleason pattern 3 would be currently assigned a pattern 5 [5, 13]. These changes in criteria, in addition to the well-recognized interobserver variability in grading among general surgical pathologists compared to pathologist with urologic pathology expertise [20, 21], must be responsible for most of our significant change in GS in the current study. The change in the overall GS was mainly driven by reassignment of the secondary pattern of Gleason grade as shown in Table 2. Our findings further emphasize the need for central pathologic review of archival specimens by subspecialty experts. The latter is crucial to ensure accuracy and applicability of any biomarker research findings that could be skewed by the great impact of potential GS variations similar to the ones found here. This latter point is highlighted in our finding of the impact of GS ISUP 2005 reassignment in our cohort on the predictive power of a genomic assay like Decipher (Table 4) where a lower, albeit statistically significant HR for Decipher was found when adjusted for the reassigned GS by expert urologic pathologist compared to original GS.

The 2005 ISUP modified Gleason system has been previously shown to be superior to the original Gleason system in predicting BCR following prostatectomy [13, 14, 22, 23]. The later has provided the premise for the recent proposal of assigning a “Prognostic Grade Group” (Groups I-V) based on ISUP 2005 GS [8, 24]. In the current study we aimed to clinically validate the ISUP 2005 modified Gleason grading system ability to predict the occurrence of clinically recurrence (metastasis), BCR and PCSM in a clinically and pathologically well annotated natural history cohort of intermediate and high risk men undergoing RP. Our findings further validate the superiority of the ISUP 2005 GS in predicting metastasis and survival outcomes. We found a superior separation of cumulative metastasis incidence curves among the reassigned 2005 ISUP GS 4+3 and GS 8 (corresponding to the above mentioned proposed Prognostic Grade Groups III and IV respectively) [Fig 4]. We also found an increased HR for the association of both GS 8 and GS 9 and incidence of metastases using the reassigned ISUP 2005 GS values compared to original GS (Table 3). The reassigned ISUP 2005 GS outperformed original GS using survival concordance index at 5 years post RP (0.80 vs. 0.70) and showed a superior net benefit on decision curve analysis across a wide range of probability thresholds from 5–30% (Fig 5).

On multivariable, extraprostatic extension and GS 8 were only significant in model 1 where all the original variables were used. In model 2, the reviewed data were used for GS, extraprostatic extension, SV invasion and surgical margins, which might lead to the drop of significance of GS 8 and extraprostatic extension.

The large size of our well annotated natural history cohort of intermediate and high risk prostate cancer patients and the central review of all specimens pathologic features by urologic pathology experts are perceived strength of our study. The retrospective nature of the study is a limitation. Another weakness of the study is that the original pathologic assessment of some cases was done by general surgical pathologists. It is therefore likely that a comprehensive re-review by urologic pathologists is at least partially responsible for the better association with outcome rather than solely the adherence to the modified ISUP 2005 GS criteria. The lack of original Gleason 6 patients due to the study design can be seen as another limitation.

In summary, we found significant difference between original and reassigned ISUP modified GS in our cohort. Upgrading was more frequently seen among the group of men with progression to metastatic disease in which almost one third of cases were upgraded. The ISUP 2005 modified GS was found to better predict the development of metastasis post RP. Strong consideration should be made for re-grading pathologic tissue when conducting retrospective biomarker studies.

## Supporting Information

S1 Fig Cumulative incidence curves of (A) BCR by original Gleason score, (B) BCR by reviewed Gleason score, C) PCSM by original Gleason score, D) PCSM by reviewed Gleason score. The number at risk in each group is shown in the footnote lines. Patients in the sub-cohort are used.
(TIF)

S2 Fig A) Survival concordance index for predicting PCSM at 10 years post radical prostatectomy. C-index of the reassigned Gleason score was higher than the original Gleason score. B) Decision curve analysis at 10 years post radical prostatectomy. The reassigned ISUP 2005 Gleason score shows a higher net benefit compared to original Gleason score.
(TIF)
