# Supplementary figures and images for: Clinical Validation of the 2005 ISUP Gleason Grading System in a Cohort of Intermediate and High Risk Men Undergoing Radical Prostatectomy
Source: PLoS One. 2016 Jan 5;11(1):e0146189. doi: 10.1371/journal.pone.0146189 (PMC4712132; doi:10.1371/journal.pone.0146189)

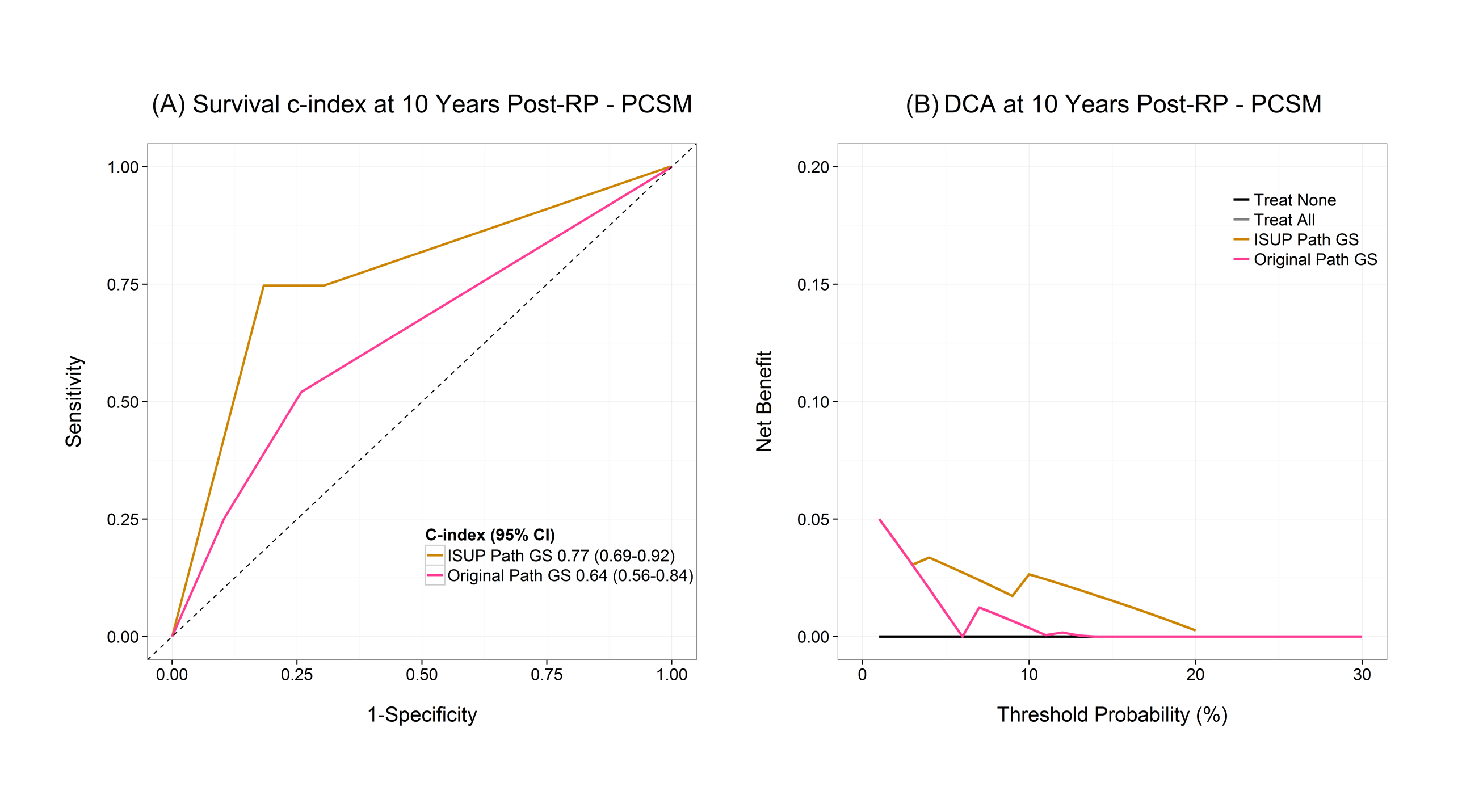

Supplement: S2 Fig — The reassigned ISUP 2005 Gleason score shows a higher net benefit compared to original Gleason score. (TIF) [file pone.0146189.s002.tif]
